# Distinctive Patterns of Seizure-Related White Matter Alterations in Right and Left Temporal Lobe Epilepsy

**DOI:** 10.3389/fneur.2019.00986

**Published:** 2019-10-01

**Authors:** Irena Buksakowska, Nikoletta Szabó, Lukáš Martinkovič, Péter Faragó, András Király, Jiří Vrána, Zsigmond Tamás Kincses, Jan Meluzín, Vlastimil Šulc, Martin Kynčl, Miloslav Roček, Michal Tichý, František Charvát, Daniel Hořínek, Petr Marusič

**Affiliations:** ^1^Department of Radiology, Second Faculty of Medicine, Charles University and Motol University Hospital, Prague, Czechia; ^2^Department of Neurology, Faculty of General Medicine, University of Szeged, Szeged, Hungary; ^3^Department of Neurology, Second Faculty of Medicine, Charles University and Motol University Hospital, Prague, Czechia; ^4^Department of Radiodiagnostics, University Central Military Hospital, Prague, Czechia; ^5^Department of Neurosurgery, Second Faculty of Medicine, Charles University and Motol University Hospital, Prague, Czechia

**Keywords:** temporal lobe epilepsy (TLE), neuroimage analysis, diffusion tensor image analysis, white matter, hippocampal sclerosis (HS)

## Abstract

**Background:** We hypothesized that right and left temporal lobe epilepsy (RTLE and LTLE, respectively) have distinctive spatial patterns of white matter (WM) changes that can be differentiated and interpreted with the use of multiple diffusion parameters. We compared the global microstructure of fiber bundles with regard to WM alterations in both RTLE and LTLE, addressing some of the methodological issues of previous studies.

**Methods:** Diffusion tensor imaging data from 17 patients with RTLE (age: 40.7 ± 10.4), 15 patients with LTLE (age: 37.3 ± 10.4), and 15 controls (age: 34.8 ± 11.2) were used in the study. WM integrity was quantified by fractional anisotropy (FA), mean diffusivity (MD), longitudinal diffusivity (LD), and radial diffusivity (RD). The diffusion parameters were compared between the groups in tracts representing the core of the fiber bundles. The volumes of hippocampi and amygdala were subsequently compared across the groups, while the data were adjusted for the effect of hippocampal sclerosis.

**Results:** Significantly reduced FA and increased MD, LD, and RD were found bilaterally over widespread brain regions in RTLE. An increase in MD and RD values was observed in widespread WM fiber bundles ipsilaterally in LTLE, largely overlapping with regions where FA was lower, while no increase in LD was observed. We also found a difference between the LTLE and RTLE groups for the right hippocampal volume (with and without adjustment for HS), whereas no significant volume differences were found between patients and controls.

**Conclusions**: It appears that patients with RTLE exhibit a more widespread pattern of WM alterations that extend far beyond the temporal lobe in both ipsilateral and contralateral hemisphere; furthermore, these changes seem to reflect more severe damage related to chronic degeneration. Conversely, more restrained changes in the LTLE may imply a pattern of less severe axonal damage, more restricted to ipsilateral hemisphere. Comprehensive finding of more prominent hippocampal atrophy in the RTLE raises an interesting issue of seizure-induced implications on gray matter and WM microstructure that may not necessarily mean a straightforward causal relationship. Further correlations of diffusion-derived metrics with neuropsychological and functional imaging measures may provide complementary information on underlying WM abnormalities with regard to functional hemispheric specialization.

## Introduction

Temporal lobe epilepsy (TLE) is the most prevalent type of focal epilepsy. Whereas, neuronal loss and gliosis in hippocampus [hippocampal sclerosis (HS)] is the most common underlying pathological finding in TLE (1), widespread gray matter abnormalities are also frequently reported, including further mesiotemporal changes that extend to the parahippocampus and entorhinal cortex (2), amygdala (3), thalamus, and multiple cortical regions. Although TLE is considered to be a gray matter disorder, changes in white matter (WM) fibers that reflect altered underlying brain connectivity seem to have important implications in terms of seizure generation and propagation (4, 5).

Consistently, it has been shown that WM changes associated with TLE are not restricted to the affected medial temporal lobe and involve a larger epileptogenic network (5, 6), reflecting underlying seizure-related WM alterations. Numerous neuroimaging studies on unilateral TLE revealed further temporal and extra-temporal WM changes (7–10).

In recent decades, a growing number of studies have emphasized the role of diffusion tensor imaging (DTI) in the assessment of microstructural WM integrity. DTI provides quantitative information about the direction and magnitude of water diffusion within tissues with the use of tensor-derived parameters: fractional anisotropy (FA), mean diffusivity (MD), diffusivity longitudinal (axial, λ1, LD), and perpendicular [radial diffusivity—RD (λ2+λ3)/2)] to the principal diffusion direction. Tract-Based Spatial Statistics (TBSS) is an automated method that allows for performing multi-subject statistical testing of diffusion-related parameters; misalignment issues are solved with restricting analysis to the core of fiber bundles, represented by the local maxima of FA (11). TBSS aims to improve the sensitivity, objectivity, and interpretability of multi-subject analysis as it combines advantages of voxel-based approaches (enabling evaluation of the whole brain without a priori predefining voxels or tracts of interest) with advantages of tractography-based approaches (estimating FA from relevant voxels). Moreover, while tractography measurements are derived from averaging values over all voxels within an individual WM tract, which can possibly exclude discrete areas of difference, TBSS can quantify DTI-based WM alterations throughout the entire brain without an a priori hypothesis, and at the same time detect different patterns of abnormality within individual tracts.

Several studies employed TBSS to evaluate WM changes in TLE patients in comparison to healthy controls (9, 12–14); however, most of the reported TLE-related WM alterations were described without consideration of the affected side or possible discrepancies resulting from lateralization of brain function. We hypothesized that right and left TLE (RTLE and LTLE, respectively) have different spatial patterns of WM changes that can be differentiated and interpreted with the use of multiple diffusion parameters. The current study compares global microstructure of fiber bundles with regard to seizure-related WM alterations in both right and left TLE, addressing some of the methodological issues of previous studies.

## Methods

### Subjects

Fifty-four patients with drug-resistant TLE, epilepsy surgery candidates, were enrolled into the study. All of them underwent standard preoperative examination in the Motol Epilepsy Center. The evaluation included clinical examination, EEG and video-EEG monitoring, neuropsychological assessment, repeated MRI examinations (including fMRI with linguistic-based tasks), and, in selected cases, functional examinations like ictal SPECT or interictal PET. When it seemed necessary, the patients underwent an intracarotid sodium amobarbital procedure and/or intracranial EEG monitoring. Electroclinical diagnosis was concordant with TLE in all the patients.

The age- and sex-matched control subjects were recruited among the healthy volunteers, with no history of neurological or psychiatric disorder. The study protocol was approved by the local institutional ethics committee and informed consent was obtained from all subjects.

From the initial number of 54 TLE patients, 22 subjects were excluded: 3 patients due to extensive structural lesions (2 for astrocytoma and 1 for prior herpetic encephalitis), 6 due to uncertain lateralization or localization of epilepsy (1 with suspected bilateral TLE, 1 with differential diagnosis frontal lobe epilepsy vs. TLE, 4 with temporo-parietal-occipital lesions), and 8 due to the low quality of images (2 with improper acquisition, 6 with motion artifacts or incomplete examination). One patient refused subsequent examinations. Additionally, we also excluded 4 patients that were not Czech native speakers. All patients had confirmed dominance of the left hemisphere for specialized linguistic operations as evaluated with a series of fMRI tests and/or Wada test. In the LTLE group, 8 patients were diagnosed with HS (53%); in the RTLE group, 10 patients were diagnosed with HS (58%) (Table 1).

**Table 1:** Characteristics of patient groups.

		**Age (years)**	**Handedness**	**Disease duration (years)**	**Seizure frequency/month**	**MRI diagnosis**
	1	26–30	Right	20	1	Amygdala hamartoma
	2	31–35	Right	31	3	HS
	3	36–40	Right	11	9	HS
	4	31–35	Left	16	3	Non-lesional
	5	41–45	Right	25	5	FCD1 T pole + amygdala
	6	26–30	Right	16	2	HS
	7	56–60	Mixed	18	3	T pole cavernoma
RTLE	8	31–35	Right	11	3	HS
	9	26–30	Right	5	12	Amygdala hamartoma
	10	51–55	Right	9	13	HS
	11	36–40	Right	9	4	Non-lesional
	12	41–45	Left	30	1	HS
	13	46–50	Right	37	6	HS
	14	46–50	Right	48	4	HS
	15	36–40	Right	30	10	HS
	16	56–60	Right	41	4	HS
	17	36–40	Right	6	6	T pole cavernoma
	1	21–25	Mixed	6	12	Temporal encephalocele
	2	36–40	Right	26	2	HS
	3	31–35	Right	4	3	HS
	4	26–30	Right	24	3	HS
	5	41–45	Right	36	16	HS
	6	46–50	Right	9	2	Hippocampal cavernoma
	7	51–55	Right	22	2	HS
LTLE	8	26–30	Right	15	6	FCD1 T pole
	9	26–30	Right	13	15	Non-lesional
	10	36–40	Right	12	8	HS
	11	51–55	Right	6	24	T pole cavernoma
	12	36–40	Right	31	20	HS
	13	36–40	Right	19	8	Non-lesional
	14	41–45	Right	4	2	Amygdala hamartoma
	15	21–25	Left	21	5	HS

Finally, DTI data from 17 patients with RTLE (age: 40.72 ± 10.38; 10 females, 7 males), 15 patients with LTLE (age: 37.33 ± 10.38; 6 females, 9 males), and 15 age-matched normal controls (age: 34.76 ± 11.16; 8 females, 7 males) were used in this study. Shapiro-Wilk tests of normality and *t* tests were performed using the Statistical Package for Social Sciences (SPSS 17 for OS X, SPSS Inc., http://www.spss.com).

### Data Acquisition

MR imaging was carried out on a 3T GE Signa HDx MR imaging system (GE Medical Systems, Milwaukee, WI) in the Central Military Hospital, Prague. All sequences were acquired using an 8-channel head coil. The MRI protocol included high-resolution head imaging T1W (FSPGR), T2W (T2 CUBE), and FLAIR (FLAIR CUBE) 3D sequences. The FSPGR sequence parameters were as follows: TR/TE = 9.33/3.88, 120 slices, slice thickness 1 mm without gap, 320 × 256 matrix with a FOV of 24 × 24 cm^2^. Diffusion tensor images were obtained with a diffusion-weighted, single-shot, echo-planar imaging (*b* = 1,000 s/m^2^ images with 30 non-collinear diffusion directions—TR: 15,000 ms, TE: 89 ms, matrix: 128 × 128, FOV: 24 × 24 cm, flip angle: 90°, in-plane resolution: 1.8 × 1.8 mm^2^, slice thickness: 2.4 mm, with 5 non-diffusion-weighted reference volumes).

### Image Analysis

DTI data were corrected for eddy currents and motion artifacts by 12 degrees of freedom (12 DOF) affine linear registration to the first non-diffusion-weighted reference image (15), which was also used to generate a binary mask with the Brain Extraction Tool implemented in FMRIB Software Library (BET, FSL v.6.0 www.fmrib.ox.ac.uk/fsl). The extraction algorithm was optimized for each subject's data. Diffusion tensors at each voxel were fitted using the FMRIB's Diffusion Toolbox (FDT). FA, MD, and LD and RD were calculated for the whole brain. In order to reduce possible errors arising from misalignment of the images, we used the Tract Based Spatial Statistics (TBSS) method (11). All subjects' FA data were aligned to the FSL FMRIB58_FA template in standard Montreal Neurological Institute (MNI) space with the use of the non-linear registration tool FNIRT (http://fsl.fmrib.ox.ac.uk/fsl/fslwiki/FNIRT), which uses a b-spline representation of the registration warp field.

A mean “FA skeleton image” was computed from the mean FA image by finding the central axis of each tract, representing the centers of all tracts common to the group. Each subject's aligned FA data were then projected onto this skeleton and thresholded at 0.2 FA. In a similar fashion, the MD, LD, and RD images were also warped to the thresholded mean FA skeleton image. The resulting data were fed into voxel-wise cross-subject statistics. Modeling and inference using standard general linear model (GLM) design setup was accomplished with the use of permutation-based cluster analysis (5,000 permutations) (16) as implemented in FSL. The regressors of the GLM analysis coded for group membership and clinical variables in the design. The regressors, age and gender, were demeaned. Correlation analysis was conducted between diffusivity parameters and seizure frequency. With the GLM design, negative and positive correlations were calculated. For statistical inference, a Threshold-Free Cluster Enhancing (TFCE) approach was used (17).

### Volumetric Analysis of the Subcortical Structures

Acquired structural images were additionally processed and automatic segmentation of hippocampi and amygdalae was carried out with FMRIB's Integrated Registration Segmentation Toolkit (FIRST) (18). Structural data were available and complete for all the LTLE patients, 16 RTLE patients and 14 controls. Volumetric comparisons were performed using the Statistical Package for Social Sciences (SPSS 25 for Windows, SPSS Inc., http://www.spss.com). The volumes of the segmented structures of hippocampi and amygdala were compared across groups (sides included) with a non-parametric Mann–Whitney *U* test.

In the next step, in order to explain the variance between groups (RTLE, LTLE and controls), we examined the influence of the diagnosed HS as a covariate on the measured hippocampal volumes. For this purpose, we used Analysis of Covariance (ANCOVA), which is a combination of an ANOVA and a regression analysis and that examines the influence of an independent variable on dependent variables, while removing the effect of the covariate factor.

## Results

### Clinical Variables

The clinical and demographic variables of patients are summarized in Table 1. No significant differences were found between the age or gender distribution of the groups (*p* > 0.05). The groups did not differ in handedness (*p* > 0.05). In regard to disease duration and seizure frequency (seizure/month), the patients' groups did not show difference (*p* > 0.05).

### Hippocampal and Amygdalar Volumetry

The size of hippocampi and amygdala was compared between groups. A total of 18 tests were conducted. Because the Kolmogorov–Smirnov test indicated that the normality assumption was violated, a non-parametric Mann–Whitney *U* test was performed.

The RTLE group of patients showed a significantly smaller size of the right hippocampi (unilateral side) compared to the left side (*p* < 0.024). The size of unilateral amygdala was also smaller, but the difference was not significant (*p* < 0.193).

It was also shown that the right hippocampi were significantly smaller when the RTLE group was compared with the LTLE. Unilateral hippocampal and amygdalar volumes were also smaller for the LTLE group, although with no significant difference (*p* < 0.290 and *p* < 0.130).

When compared with the controls, the only statistically significant result was shown for amygdalar volumes in LTLE (*p* < 0.023).

**Figure 1:**
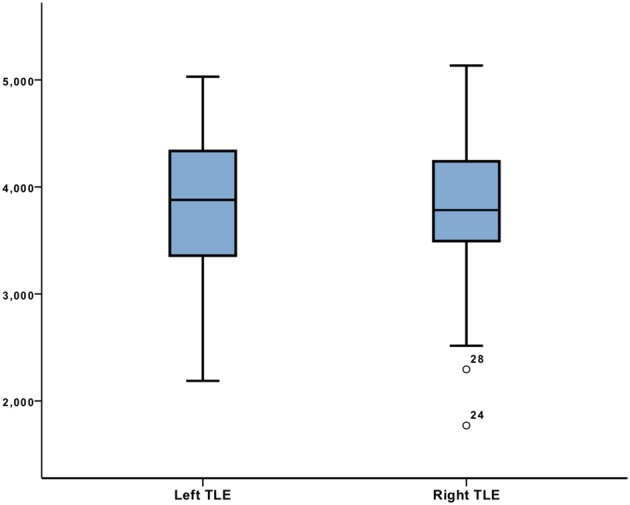
Right hippocampal volume comparison between left TLE and right TLE adjusted for HS with ANCOVA.

These results were further confirmed with the analysis of covariance. While the data were adjusted for the effect of HS (roughly more than half of subjects in patient's groups), the difference between the LTLE and RTLE groups for the right hippocampal volume remained the only statistically significant finding (*p* < 0.014; Figure 1) and Supplementary Figure 1.

No significant differences were found between the patients and controls.

### WM Microstructure Alterations in Patients With RTLE Compared to Controls

**Figure 2:**
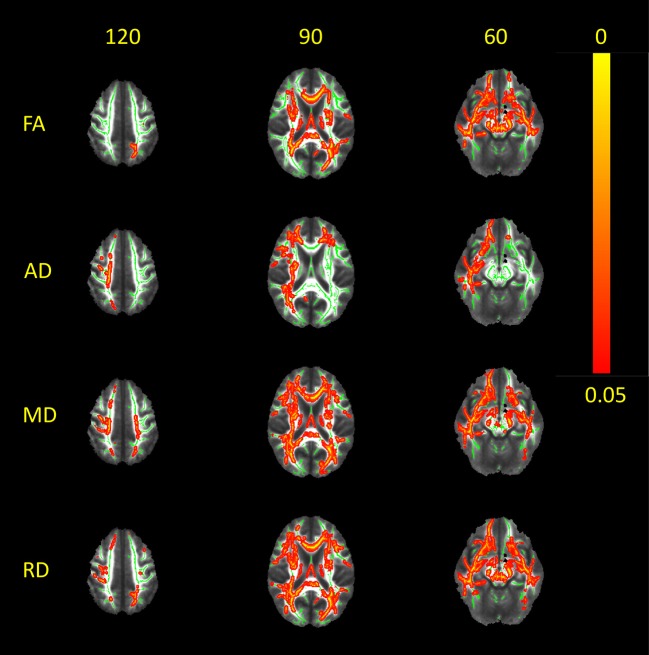
White matter alterations in RTLE as compared to normal controls. Red-yellow clusters indicate statistically significant results in patients: widespread clusters of reduced FA as depicted in the center of fiber bundles, paralleled with increased MD, RD, and AD. It appears that patients with RTLE exhibit a large-scale pattern of WM alterations that extends beyond the temporal lobe. Significant clusters were thickened in order to obtain better visualization. Results are overlaid on the mean FA image; the WM skeleton thresholded at 0.2 is shown in green. Images are thresholded at *p* < 0.05, corrected for multiple comparisons. MD, mean diffusivity; AD, axial/longitudinal diffusivity; RD, radial/perpendicular diffusivity.

Significantly reduced FA (*p* < 0.01) was found bilaterally over widespread brain regions in RTLE patients when compared to the controls (Figure 2), including the genu and body of corpus callosum (but not splenium), fornix and posterior limb of internal (and external) capsule. Reduced FA values were more widely distributed in the ipsilateral hemisphere in the inferior fronto-occipital fasciculus, uncinate fasciculus, inferior longitudinal fasciculus, and caudal part of forceps minor; the largest changes were seen in the WM of the right temporal pole and the WM close to the temporal and occipital fusiform cortex and parahippocampal gyrus. Additionally, reduced FA values were present in the contralateral superior longitudinal fasciculus, superior and posterior part of the corona radiata, and cranial parts of the forceps minor.

Increased MD (*p* < 0.02) was widespread in the RTLE patients, highly overlapping with the FA changes. Again, an increase of MD was predominantly seen in the ipsilateral hemisphere: in the inferior fronto-occipital fasciculus, uncinate fasciculus, and inferior longitudinal fasciculus. Moreover, higher MD values in the superior longitudinal fasciculus, superior and posterior parts of the corona radiata, and the forceps minor were also essentially ipsilateral. Only some fiber bundles (the forceps major and the posterior part of the inferior fronto-occipital fasciculus) showed a predominantly contralateral MD increase.

RD was also higher (*p* < 0.01) in the RTLE patients as compared to the controls over the widespread WM fiber bundles, mostly ipsilaterally. Regions with significantly higher RD included WM fibers arising from the frontal and parietal cortex passing through the posterior limb of the internal capsule, superior longitudinal fasciculus, inferior longitudinal fasciculus, inferior fronto-occipital fasciculus (mostly the anterior part), uncinate fasciculus, and forceps minor. Whereas, generally diffuse, these changes were mostly presented in the right hemisphere.

Again, solitary clusters in the posterior part of the left inferior fronto-occipital fasciculus/forceps major were more noticeable contralaterally. Whereas, there were higher values in the bilateral body and genu of the CC (less in the splenium), no RD changes were shown in fornix.

Finally, higher values of LD (*p* < 0.02) were found exclusively (or almost exclusively) in the ipsilateral inferior longitudinal fasciculus, uncinate fasciculus, superior longitudinal fasciculus, and posterior limb of internal capsule; minor changes in the genu and body of the CC were seen bilaterally.

### WM Microstructure Alterations in Patients With LTLE Compared to Controls

**Figure 3:**
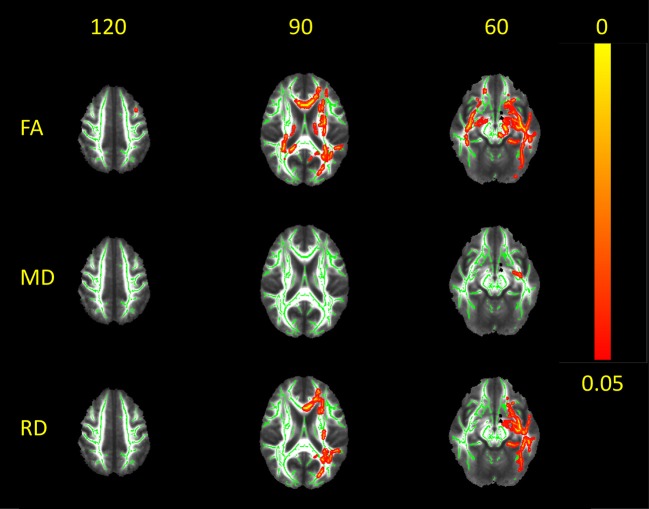
White matter alterations in LTLE as compared to normal controls. Red-yellow clusters indicate statistically significant results in patients: clusters of reduced FA and increased RD in the center of main fiber bundles (along with focal clusters of increased MD) are more restricted to the ipsilateral hemisphere. Significant clusters were thickened in order to obtain better visualization. Results are overlaid on the mean FA image; the WM skeleton thresholded at 0.2 is shown in green. Images are thresholded at *p* < 0.05, corrected for multiple comparisons.

FA was reduced in the patients with LTLE compared to the controls over the WM fiber bundles in the ipsilateral hemisphere, most prominently in the left uncinate fasciculus and inferior fronto-occipital fasciculus (*p* < 0.01; Figure 3). Additional WM regions with significantly lower FA (*p* < 0.02) were also found in the ipsilateral inferior longitudinal fasciculus, superior longitudinal fasciculus, forceps minor and forceps major, and body and genu of the corpus callosum. Furthermore, some smaller clusters also appeared in the contralateral inferior fronto-occipital fasciculus and uncinate fasciculus.

Increased MD and RD values (*p* < 0.02) were observed in widespread WM fiber bundles ipsilaterally, largely overlapping with regions where FA was lower (inferior longitudinal fasciculus, uncinate fasciculus, inferior fronto-occipital fasciculus, and superior longitudinal fasciculus). Moreover, MD was higher in ipsilateral corona radiata; small clusters of higher MD were also found in the genu and body of the corpus callosum. No higher MD values were seen contralaterally, and only some clusters of higher perpendicular diffusivity were shown in the right superior and inferior longitudinal fasciculus, when the threshold was lower (*p* < 0.05).

No LD increase was observed in the patients with LTLE compared to the controls.

### WM Microstructure Alterations in Patients With LTLE Compared to RTLE

No difference was found between the left and right TLE groups.

## Discussion

Group-level analyses of multiple diffusion parameters in the core of WM fiber bundles showed different patterns of WM changes in the RTLE and LTLE as compared to the controls. Whereas, LTLE-related WM alterations were predominately or exclusively ipsilateral, temporal and extra-temporal WM abnormalities in the RTLE appeared to be more widespread and bilateral (although also predominantly ipsilateral).

As expected, widespread WM changes were most pronounced in pathways terminating in the temporal lobe (including the inferior longitudinal fasciculus, uncinate fasciculus, and superior longitudinal fasciculus). Our findings are consistent with previous DTI studies that showed TLE-related WM abnormalities that are not only confined to the temporal lobe but associated with a larger epileptogenic network (7, 10, 19–22).

Some previous TBSS studies either combined the image data from both left and right TLE (9, 10, 14, 23, 24) or mirrored RTLE images across the midline (19, 23), thus adding more anatomical variances and making group differences more difficult to detect (19). We solved these issues by keeping left and right TLE data distinct and comparing them separately with age- and sex-matched controls in order to further differentiate WM abnormalities depending on the hemisphere of the seizure onset.

Most of the previous TBSS studies demonstrated TLE-related WM aberrations as illustrated solely by either FA reduction (21, 23) or FA reduction followed by MD increase (9, 25). In our study, we used multiple DTI metrics to provide complementary information about the underlying WM seizure-related WM damage and to strengthen the comprehensibility of the results.

In general, high MD values indicate reduced axonal fiber caliber and increased extracellular space. Whereas, FA values reflect the underlying integrity of WM tracts, MD values can also indicate the structural changes in gray matter neurons (10). The increased longitudinal diffusivity can be explained by increased extra-axonal space due to reduced axonal density and greater diffusion parallel to axons (26) as a result of axonal damage and degeneration (secondary to Wallerian degeneration), whereas diffusivity perpendicular to *the principal diffusion direction* mainly reflects the degree of demyelination (27, 28).

The pattern that we observed in LTLE with ipsilaterally decreased FA and increased perpendicular diffusivity, accompanied by non-significant change in longitudinal diffusivity, may be suggestive of earlier or less severe stages of degeneration (29, 30).

Conversely, severe and extensive WM microstructural alterations in RTLE represented by reduced FA and higher values of MD, LD, and RD in temporal and extratemporal WM bundles suggest an underlying combination of axon and myelin loss and indicate more severe tract damage related to advanced stages of Wallerian degeneration, likely as a result of cortical and subcortical gray matter pathology. Hippocampal volumetric measures confirmed these results, suggesting that damage of both white and gray matter was more severe in the RTLE group, as addressed later in discussion.

Previous studies have confirmed that WM structural integrity in TLE is thought to be disturbed more severely in the ipsilateral rather than contralateral hemisphere, as revealed by FA and MD changes (5, 10, 31, 32). Usually, fiber bundles connected with the affected temporal lobe are the ones most severely altered (31) and showing a centrifugal pattern of changes. Similarly, asymmetry of WM integrity (explored in terms of lower FA values) was found to be associated with a leftward or rightward tendency, depending on the affected hemisphere (33). Interestingly, some studies indicated that TLE patients with left hemispheric onset exhibit more alterations in ipsilateral and contralateral regions than those with RTLE, manifested as FA reduction and/or MD increase in temporal and extra-temporal fiber bundles (20, 34, 35); these findings were also confirmed postoperatively (21). Other studies on the effect of hemispheric laterality on diffusion parameters revealed more widespread WM abnormalities in RTLE patients than in those with LTLE (22, 36). It can be concluded that the diverse distribution of WM alterations suggests that the localization of epileptic networks may play a role in the WM burden, regardless of which initial side is studied (37).

Several possible explanations have been proposed to resolve the hemispheric predominance of WM changes. It has been suggested that neuronal connections in either the left or right hemisphere, respectively, may be more likely to support seizure propagation to the contralateral hemisphere (34, 35). In particular, it is possible that seizures cause more excitotoxic damage while originating in the dominant hemisphere, whereas the brain tissue in the dominant hemisphere may be more susceptible to pathological impact (35). In addition, the role of different maturation speed between both hemispheres has been proposed as another potential explanation for these asymmetries, implying that neurodevelopmental factors may play an important role in the epileptogenic process of TLE (38).

Importantly, many discrepancies between laterality of findings may be explained by the heterogeneity in the studied populations, followed by differences in analytical approaches. Overall, a growing corpus of research demonstrates the strong effect of etiologically distinct and pathologically different syndromes underlying TLE, while mesiotemporal–hippocampal sclerosis (HS) is considered to be a distinct clinico-pathologic entity from a non-HS group that appears to be highly heterogeneous (39).

Our results, suggesting that the epileptogenic network appears to be larger in RTLE, are partially consistent with findings presented by Bonilha et al. (36) that identified additional MD changes expressed in RTLE (additionally to diffuse pattern of FA changes in both LTLE and RTLE) and with findings described by Oguz et al. (32) that showed more extensive MD changes in RTLE in groups of both female and male patients. Also, the results described by Lemkaddem et al. are broadly consistent with the evidence that bilateral neocortical networks are severely affected in RTLE; the extensive pattern of alterations affecting temporal and extratemporal structures has been shown with a more complex combination of microstructural changes (22).

Our study confirmed more restricted WM abnormalities in LTLE than in RTLE as compared to controls; the diffusion-related asymmetry was thus more obvious in the LTLE group, with WM alterations found predominantly or almost exclusively ipsilaterally. The number of significant voxels was considerably larger in the contralateral hemisphere in RTLE subjects. Also, hippocampal volumes were significantly smaller ipsilaterally in the RTLE group, although significant analogous results were not confirmed in the LTLE group.

As already implied, several factors may influence the degree and extent of diffusion alterations in TLE, including the severity of mesiotemporal lobe sclerosis (neuronal loss and gliosis) (5, 23). Our results seem to partially confirm that widespread and severe diffusion abnormalities were more apparent in the group with significant ipsilateral hippocampal volume reduction. Contrary to studies that did not detail the repartition of HS vs. non-HS findings on MRI, we examined the influence of HS on the measured volumes, given the fact that more than half of the patients in each group demonstrated HS with a similar rate. Our analyses confirmed the diagnostic role of HS as a covariate when explaining the variance between RTLE and LTLE, but no volume differences were found in comparison to the controls. Our primary objective was to assess the degree of severity of hippocampal atrophic rates in both right and left TLE, and the diagnostic partition appeared to be consistent with these results. Our results might therefore be dominated to some extent by the finding of more prominent hippocampal atrophy in the RTLE group; however, these findings may not necessarily imply a causal relationship.

Whereas, a growing number of studies imply that the degree of WM alterations appears to be heavily influenced by the side of seizure onset and macroscopic structural changes, converse evidence from animal models and human studies suggests that hippocampal neural loss can occur following severe or prolonged seizure activity (40). Some authors suggest that patients who experience secondary generalized seizures are more prone to contralateral hippocampal volume loss (41). Others, on the other hand, argue against the hypothesis that epileptiform activity *per se* contributes to focal brain injury in previously undamaged cortical regions (42) and imply that structural brain damage is not an inevitable consequence of epileptic seizures (43).

Likewise, early presentation of diffusion abnormalities despite the lack of volumetric changes (44) would be indicative of contributing factors of neurodevelopmental nature. It is also possible that the microstructural architecture of WM can be at least partially altered by the presence of developmental neuronal remnants (interstitial neurons) that have been more recently reported in epilepsy (5). These findings may be interpreted as arrested neuronal migration and/or increased WM neurogenesis from progenitor cells (45, 46).

One important limitation of our study is that the time between the last seizure and MR scan was unknown and thus interictal heterogeneity within the patient groups was possible. There is growing evidence of postictal changes that may persist for longer periods of time in some patients, and which are reflected as a decrease in diffusivity (47). Specifically, these changes likely indicate cellular swelling in the area of seizure onset and possibly areas of seizure spread. Further systematic studies may therefore shed more light on complex and dynamic changes that reflect the timing of seizure and imaging. Special focus on the correlation of these changes with outcome after epileptic surgery may be the key component to determining the role of postictal diffusion measures in the presurgical evaluation of epilepsy patients.

Moreover, due to recent findings suggesting that WM architectural changes may be reversible in the contralateral hemisphere after a successful surgery in TLE (48), it would also be interesting to perform analyses on pre- and post-postoperative data, as it may help differentiate the impairment attributed either to the outcome of resection or to underlying seizure disorder. Also, further correlations of diffusion-derived metrics with quantified neuropsychological and functional imaging findings may provide complementary information about the underlying seizure-related WM abnormalities with regard to functional hemispheric specialization.

## Conclusions

Overall, our results are broadly consistent with studies confirming that patients with TLE suffer from dysfunctions affecting large-scale brain networks rather than a single focal region. Our study implies that right and left TLE have different distinctive spatial patterns of WM microstructural abnormalities that can be differentiated and interpreted with the use of multiple diffusion metrics. It appears that patients with RTLE exhibit a more widespread pattern of WM alterations that extends far beyond the temporal lobe in both the ipsilateral and contralateral hemisphere; furthermore, these changes seem to reflect more severe damage related to chronic degeneration. On the other hand, diffusion changes in LTLE may suggest a pattern of less severe axonal damage, more restricted to ipsilateral hemisphere. Partially comprehensive finding of more prominent hippocampal atrophy in the RTLE raises an interesting issue of seizure-induced implications on gray matter and WM microstructure that may not necessarily mean a straightforward causal relationship. Additional correlations of diffusion-derived metrics with neuropsychological and functional imaging measures may provide complementary information about underlying seizure-related WM abnormalities with regard to functional hemispheric specialization, and correlations with outcome after epileptic surgery may determine the role of postictal diffusion measures in the presurgical evaluation of TLE patients. An interesting issue to address further would be whether these abnormalities might be reversible with good seizure control or surgery.

## Data Availability Statement

The datasets analyzed in this manuscript are not publicly available. Requests to access the datasets should be directed to IB (irena.buksakowska@gmail.com).

## Ethics Statement

The studies involving human participants were reviewed and approved by Second Faculty of Medicine, Charles University and Motol University Hospital, Prague, Czech Republic. The patients/participants provided their written informed consent to participate in this study.

## Author's Note

Preliminary and partial results were presented previously in conference proceedings: IB, LM, NS, JV, ZK, JM, VŠ, Amlerova J., Kyncl M., Rocek M., Horinek D., Charvat F., Marusic P., *Distinctive patterns of seizure-induced white matter damage in right and left temporal lobe epilepsy*, 12th European Congress on Epileptology, Prague 2016.

## Author Contributions

All authors listed have made a substantial, direct and intellectual contribution to the work, and approved it for publication.

### Conflict of Interest

The authors declare that the research was conducted in the absence of any commercial or financial relationships that could be construed as a potential conflict of interest.

## Supplementary Material

Supplementary Figure 1 While the data were adjusted for the effect of HS the difference between the RTLE and LTLE groups for the right hippocampal volume remained the only statistically significant finding.
