# Supplementary figures and images for: Distinctive Patterns of Seizure-Related White Matter Alterations in Right and Left Temporal Lobe Epilepsy
Source: Front Neurol. 2019 Oct 1;10:986. doi: 10.3389/fneur.2019.00986 (PMC6779711; doi:10.3389/fneur.2019.00986)

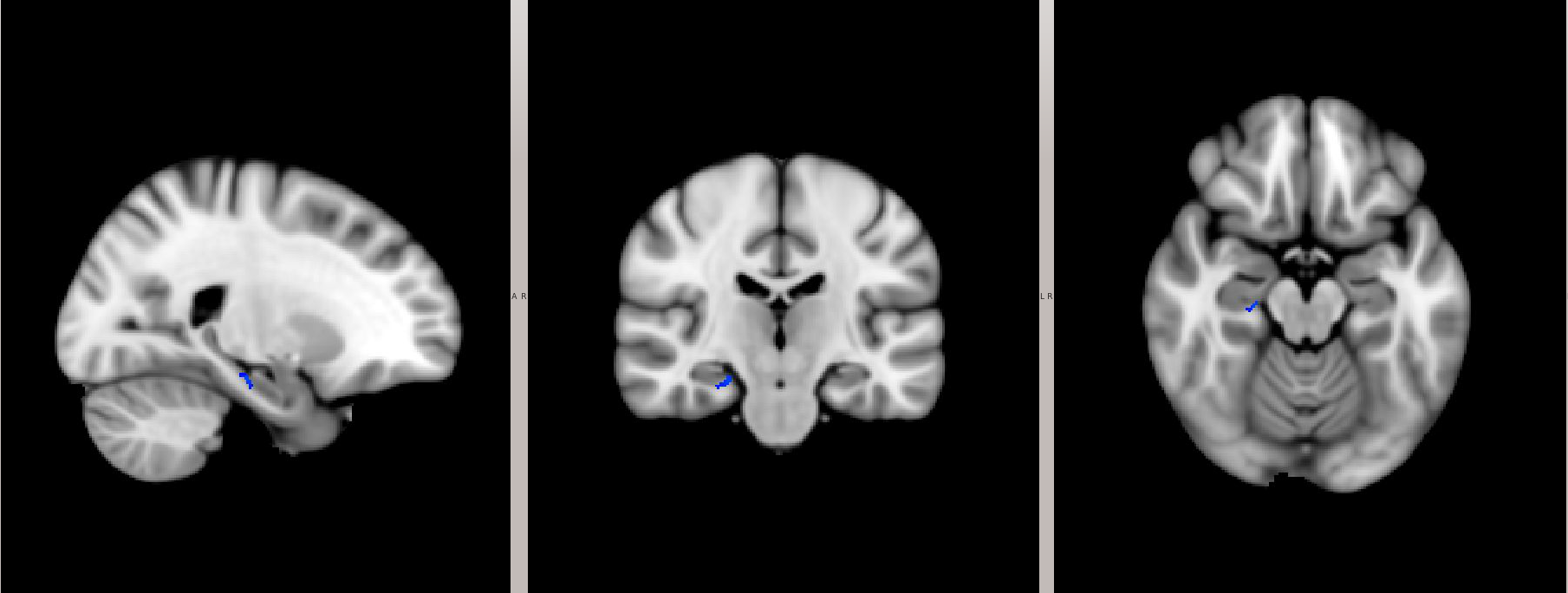

Supplement: Supplementary Figure 1 — While the data were adjusted for the effect of HS the difference between the RTLE and LTLE groups for the right hippocampal volume remained the only statistically significant finding. [file Image_1.TIFF]
